# Pancreatic Perivascular Epithelioid Cell Tumour Presenting with Upper Gastrointestinal Bleeding

**DOI:** 10.1155/2015/431215

**Published:** 2015-01-06

**Authors:** Christos Petrides, Kyriakos Neofytou, Aamir Z. Khan

**Affiliations:** ^1^Department of Surgery, Nicosia Government Hospital, Palaios Dromos Lefkosias-Lemesou, No. 215, Strovolos, 2029 Nicosia, Cyprus; ^2^Department of Academic Surgery, Royal Marsden Hospital, Upper GI/HPB Unit, Fulham Road, London SW3 6JJ, UK

## Abstract

PEComa is a family of rare mesenchymal tumours which can occur in any part of the human body. Primary PEComas of the pancreas are extremely rare tumours with uncertain malignant potential. A 17-year-old female was admitted to the hospital due to melena. She required several transfusions. CT scan demonstrated a mass at the head of the pancreas measuring 4.2 cm in maximum diameter. An endoscopic ultrasound showed an ulcerating malignant looking mass infiltrating 50% of the wall of the second part of the duodenum in the region of the ampulla. Multiple biopsies taken showed extensive ulceration with granulation tissue formation and underlying large macrophages without being able to establish a definite diagnosis. We proceeded with pylorus-preserving pancreaticoduodenectomy. The postoperative course of the patient was unremarkable, and she was discharged on the 8th postoperative day. Histology examination of the specimen showed a PEComa of pancreas. Eighteen months after resection the patient is disease free. To the best of our knowledge this is the first time we describe a case of a pancreatic PEComa presenting with massive gastrointestinal bleeding.

## 1. Introduction

PEComa (perivascular epithelioid cell tumour) is a family of mesenchymal tumours consisting of perivascular epithelioid cells (PECs). PEComas are rare tumours that can occur in any part of the human body [1]. The most common tumors in the PEComa family are renal angiomyolipoma and pulmonary lymphangioleiomyomatosis, both of which are more common in patients with tuberous sclerosis complex [1–3]. Establishing the malignant potential of PEComas remains challenging although criteria have been suggested [1–3].

Primary PEComas of the pancreas are extremely rare tumors with uncertain malignant potential. Only twelve cases, including the one we report here, are published in the literature [2, 4–13]. Surgical resection represents the only curative approach for this kind of tumours [1, 2, 4–13].

Here, we present a rare case of a patient with upper gastrointestinal bleeding due to an ulcerating head of pancreas PEComa. This patient underwent PPPD and 18 months after operation is disease free. To the best of our knowledge this is the first report of PEComa of pancreas manifesting with upper gastrointestinal bleeding.

## 2. Case Report

**Figure 1:**
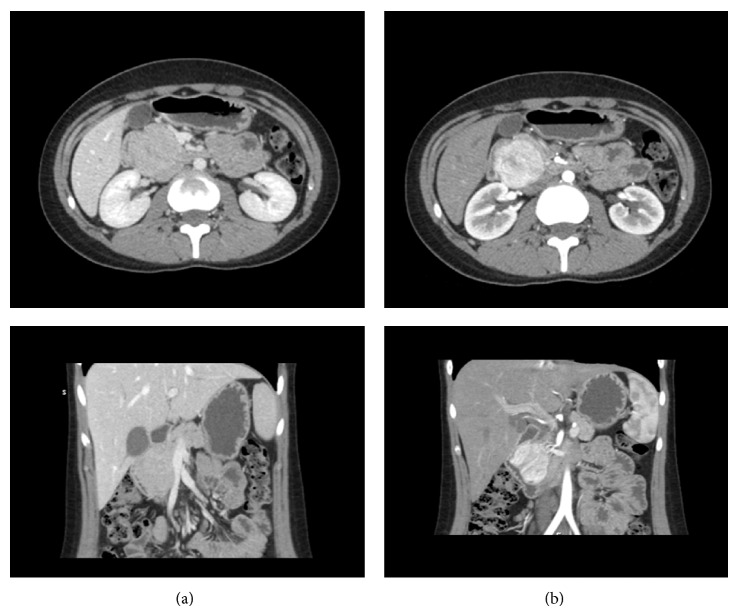
Abdomen computed tomography showing a mass at the head of pancreas. (a) Portal vein phase: isodense appearance of the mass to the rest of the pancreas. (b) Arterial phase: enhancement of the mass.

A 17-year-old female patient was referred to RMH due to melena caused by a mass at the head of pancreas. She presented at the local hospital 2 months before with melena. At that time she required several transfusions due to anemia (hemoglobin 6 g/dL at presentation) and she underwent oesophagogastroduodenoscopy (OGD), colonoscopy, and Meckel's scan; all of them reported as normal. A CT scan revealed a mass at the head of pancreas (Figure 1).

At the time of referral she was asymptomatic. Her past medical history and the clinical examination were unremarkable. The review of the CT scan, which took place at the local hospital, demonstrated a lesion mass at the head of the pancreas measuring 4.2 cm in maximum axial diameter and 4.9 cm in the craniocaudal direction. This mass showed avid arterial phase enhancement with rapid washout, while it appeared almost isodense compared to the rest of the pancreas in the portal venous phase. Both pancreatic duct and common bile duct were prominent, with their diameter to upper normal limit. The SMV was abutment but not involvement. Extrapancreatic disease was excluded. These features were consistent with neoplasmatic mass of the head of pancreas, with the most possible pathology being a neuroendocrine tumour. The subsequent gut hormone test was normal (VIP, PP, gastrin, glucagon, somatostatin, chromogranin A, and chromogranin B).

An endoscopic ultrasound (EUS) was performed showing an ulcerating malignant looking mass infiltrating 50% of the wall of the second part of the duodenum in the region of the ampulla. Multiple biopsies taken showed extensive ulceration with granulation tissue formation and underlying large macrophages without being able to establish a definite diagnosis.

We proceeded with pylorus-preserving pancreaticoduodenectomy. The postoperative course of the patient was unremarkable, and she was discharged on the 8th postoperative day.

Histology examination of the specimen showed an ulcerated tumour that had an expansible margin surrounded by a fibrous pseudocapsule. The tumor was well vascularised and composed of large mainly epithelioid cells with clear granular or feathery cytoplasm. Some cells with more spindle appearance were seen. The nuclei were eccentric, and there were many vascular spaces within the tumor which were dilated and some had irregular outlines. Mitosis was infrequent and there was no necrosis. No vascular invasion was seen. The cells were positive stained for HMB45, Melan A and smooth muscle actin. They were negative for cytokeratin, chromogranin, CD56, S100, desmin, and calponin. The above features were consistent with a perivascular epithelioid cell neoplasm (PEComa). Peripancreatic lymph nodes were negative for tumor and the resection was complete. Eighteen months after resection the patient is disease free.

## 3. Discussion

The perivascular epithelioid cell was first described in 1943 by Apitz as an “abnormal myoblast” in renal angiomyolipoma [14]. Since the first report of a perivascular epithelioid “sugar” tumor in the pancreas from Zamboni et al. in 1996, a number of similar lesions have been described in virtually every anatomic site of the human body [2, 3, 15–19]. These tumors can arise in patients of any age, with gender difference due to female predominance (7 : 1) [3].

PEComas are well-circumscribed and set apart from the surrounding parenchyma by a thin capsule [20]. Histopathological examination of PEComas reveals nests and sheets of usually epithelioid but occasionally spindled cells with clear to granular eosinophilic cytoplasm, often found in close association with the blood vessel walls. The tumors demonstrate immunoreactivity for both melanocytic (HMB-45, melan-A, and microphthalmia transcription factor) and smooth muscle (actin and/or desmin) markers. The term “sugar tumors” refers to the clear cytoplasm of the perivascular epithelioid cells which is rich in glycogen [15, 20].

For the most part, PEComas are considered benign; however, a subset of PEComas behaves in a malignant fashion, leading to local invasion, multiple metastases, and death as observed with high-grade sarcomas. Recently, Folpe and Kwiatkowski have suggested criteria for malignancy, including a size greater than 5 cm, mitotic count of more than 1 per 50 high-power fields, and necrosis [21]. Primary PEComas of the pancreas are extremely rare tumours with uncertain malignant potential. In the last fifteen years only twelve cases, including the one we report here, were published in the literature (Table 1). This patient group included ten women and two men (ratio 5 : 1) with a mean age of 48 years (age range from 17 to 74). Our patient is the youngest. Symptoms at diagnosis included abdominal pain in seven patients, diarrhea in one patient, a bulge in the right upper quadrant in one patient, and unspecific cold-like symptoms (fever, coughing, and fatigue) in one patient. One patient was asymptomatic, and PEComa was diagnosed using abdominal ultrasound. The tumors were located in the pancreatic head in five patients, in the pancreatic body in five patients, and in the uncinate process in one patient. The mean tumor diameter was 36 mm (range 15 to 100 mm). Tumor rupture was found only in our patient.

Our patient had a relatively small tumor (4,2 cm × 4,9 cm). Mitosis was rare and no necrosis was seen; thus Folpe's criteria for malignancy are negative for our patient. Most PEComas present with abdominal pain but our patient's first symptom was melena.

Radiographics findings with CT and MRI show that these lesions are significantly and heterogeneously enhanced on arterial phase, less enhanced on portal venous phase, and slightly hypodense on delayed phase [22]. Early recognition of pancreatic PEComas on imaging could dramatically impact both patient therapy and prognosis. Radiologists should be on high suspicion and consider the diagnosis of pancreatic PEComa if they encounter a well-defined, encapsulated, and hypovascular pancreatic mass [22].

Surgical resection represents the only curative approach for primary PEComa at presentation as well as for local recurrences and metastasis, as chemotherapy and radiotherapy have not demonstrated significant benefits [23]. Only recently limited clinical studies have reported encouraging results in terms of therapeutic response after oral administration of mTOR inhibitor in patients with metastatic PEComa [24]. Further study is warranted to determine the optimal management of these rare tumors.

## 4. Conclusions

Pancreatic PEComas are rare entities which most commonly present with abdominal pain. However, this case shows that these tumours can manifest with upper gastrointestinal bleeding.

## Figures and Tables

**Table 1:** Reported cases of pancreas PEComas and symptoms at diagnosis.

	Case	Sex	Age	Position	Size (mm)	Symptoms
1	Zamboni et al. (1996) [2]	F	60	Body	20	Abdominal pain
2	Heywood et al. (2004) [4]	F	74	Head	45	Abdominal pain
3	Ramuz et al. (2005) [5]	F	31	Body	15	Abdominal pain
4	Périgny et al. (2008) [6]	F	46	Body	17	Diarrhea
5	Hirabayashi et al. (2009) [7]	F	47	Head	17	Abdominal pain
6	Baez et al. (2009) [8]	F	60	Body	32	Bulge in right upper quadrant
7	Zemet et al. (2011) [9]	M	49	Head	32	Fever, cough, and malaise
8	Nagata et al. (2011) [10]	M	52	Head	40	Abdominal pain
9	Finzi et al. (2012) [11]	F	62	Head	25	No symptoms
10	Al-Haddad et al. (2013) [12]	F	38	Uncinate process	18	Abdominal pain
11	Okuwaki et al. (2013) [13]	F	43	Body	100	Abdominal pain
12	Our patient	F	17	Head	42	Melena

	12 patients	10F/2M	Mean age 48 (range 17–74)	Body: 5 Head: 6 Uncinate process: 1	Mean 36 mm (range 15–100 mm)	**Abdominal pain:** 7 **Bulge:** 1 **Nonspecific** **symptoms:** 2 **GI** **bleeding:** 1 **No symptoms:** 1
